# Membrane Complexes of *Syntrophomonas wolfei* Involved in Syntrophic Butyrate Degradation and Hydrogen Formation

**DOI:** 10.3389/fmicb.2016.01795

**Published:** 2016-11-09

**Authors:** Bryan R. Crable, Jessica R. Sieber, Xinwei Mao, Lisa Alvarez-Cohen, Robert Gunsalus, Rachel R. Ogorzalek Loo, Hong Nguyen, Michael J. McInerney

**Affiliations:** ^1^Department of Microbiology and Plant Biology, University of Oklahoma, NormanOK, USA; ^2^Department of Civil and Environmental Engineering, University of California, Berkeley, BerkeleyCA, USA; ^3^Department of Microbiology, Immunology, and Molecular Genetics, University of California, Los Angeles, Los AngelesCA, USA; ^4^Department of Biological Chemistry, University of California, Los Angeles, Los AngelesCA, USA

**Keywords:** syntrophy, methanogenesis, biohydrogen, hydrogenase, fatty acids

## Abstract

Syntrophic butyrate metabolism involves the thermodynamically unfavorable production of hydrogen and/or formate from the high potential electron donor, butyryl-CoA. Such redox reactions can occur only with energy input by a process called reverse electron transfer. Previous studies have demonstrated that hydrogen production from butyrate requires the presence of a proton gradient, but the biochemical machinery involved has not been clearly elucidated. In this study, the gene and enzyme systems involved in reverse electron transfer by *Syntrophomonas wolfei* were investigated using proteomic and gene expression approaches. *S. wolfei* was grown in co-culture with *Methanospirillum hungatei* or *Dehalococcoides mccartyi* under conditions requiring reverse electron transfer and compared to both axenic *S. wolfei* cultures and co-cultures grown in conditions that do not require reverse electron transfer. Blue native gel analysis of membranes solubilized from syntrophically grown cells revealed the presence of a membrane-bound hydrogenase, Hyd2, which exhibited hydrogenase activity during in gel assays. Bands containing a putative iron-sulfur (FeS) oxidoreductase were detected in membranes of crotonate-grown and butyrate grown *S. wolfei* cells. The genes for the corresponding hydrogenase subunits, *hyd2ABC*, were differentially expressed at higher levels during syntrophic butyrate growth when compared to growth on crotonate. The expression of the FeS oxidoreductase gene increased when *S. wolfei* was grown with *M. hungatei*. Additional membrane-associated proteins detected included F_o_F_1_ ATP synthase subunits and several membrane transporters that may aid syntrophic growth. Furthermore, syntrophic butyrate metabolism can proceed exclusively by interspecies hydrogen transfer, as demonstrated by growth with *D. mccartyi*, which is unable to use formate. These results argue for the importance of Hyd2 and FeS oxidoreductase in reverse electron transfer during syntrophic butyrate degradation.

## Introduction

Syntrophy is a thermodynamically based metabolic coupling between two or more microorganisms. A syntrophic partnership sustains energy production and growth for all members under conditions where no organism can manage alone. Syntrophic associations are ubiquitous in nature and essential for the complete mineralization of complex organic material to methane and carbon dioxide in natural as well as man-made environments. The degradation of key anaerobic food chain intermediates such as fatty and aromatic acids to methanogenic substrates (e.g., formate, hydrogen and acetate) is unfavorable without the methanogenic partner to maintain the low levels of the formate and/or hydrogen produced (McInerney and Bryant, 1981; Dong and Stams, 1995; Schink, 1997; McInerney et al., 2008; Sieber et al., 2012). Alternatively, syntrophic metabolism can be achieved by direct transfer via nanowires between the syntrophic partners as demonstrated, for example, in *Geobacter metallireducens* (Leang et al., 2010; Qian et al., 2011) and *Shewanella oneidensis* strain MR-1 (Gorby et al., 2006; El-Naggar et al., 2010).

The *Syntrophomonas wolfei* and *Methanospirillum hungatei* co-culture serves as a model system to study syntrophic fatty acid oxidation (McInerney et al., 1979, 1981; Müller et al., 2009; Sieber et al., 2010, 2015; Schmidt et al., 2013; Gunsalus et al., 2016). In co-culture with *M. hungatei, S. wolfei* syntrophically metabolizes short chain fatty acids of four to eight carbon atoms to acetate, using the beta-oxidation pathway (McInerney et al., 1979, 1981; Müller et al., 2009; Sieber et al., 2010, 2015; Schmidt et al., 2013). *S. wolfei* can grow in axenic culture on unsaturated fatty acids such as crotonate (Beaty and McInerney, 1987). Beta-oxidation of fatty acids generates NADH and reduced electron transfer flavoprotein (Etf), which must be reoxidized by hydrogen or formate production (Müller et al., 2009; Sieber et al., 2012, 2015; Schmidt et al., 2013). Hydrogen or formate production from NADH is favorable at concentrations maintained in methanogenic co-cultures (Schink, 1997). However, hydrogen and formate production from electrons derived from the oxidation of acyl-CoA intermediates requires energy input by a process called reverse electron transfer even at low hydrogen or formate concentrations (Schink, 1997; Sieber et al., 2012). Wallrabenstein and Schink (1994) showed that hydrogen production from butyrate by cell suspensions of *S. wolfei* required chemiosmotic energy consistent with the involvement of reverse electron transfer.

Analysis of *S. wolfei* genome revealed a membrane-bound iron-sulfur protein (SWOL_RS03525 gene product) that may act as the membrane input module for electron flow between acyl-CoA dehydrogenase and membrane redox carriers (Sieber et al., 2010; Schmidt et al., 2013). EtfAB2 and the SWOL_RS03525 gene product were abundant in the *S. wolfei* proteome, suggesting that these two enzymes may be the main conduit of electron flow between acyl-CoA dehydrogenases and membrane redox carriers (Schmidt et al., 2013; Sieber et al., 2015). In addition, the SWOL_RS03525 gene product was detected in highly purified preparations of butyryl-CoA dehydrogenase (Bcd; Müller et al., 2009), consistent with a close interaction between the SWOL_RS03525 gene product and Bcd. Peptides of a membrane-bound formate dehydrogenase (Fdh2; Schmidt et al., 2013) and transcripts of genes for a predicted, membrane-bound hydrogenase (*hyd2A*; Sieber et al., 2014) were high in butyrate-grown *S. wolfei* cells. The above data are consistent with the operation of a quinone loop involving the SWOL_RS03525 gene product and a membrane-bound hydrogenase or a formate dehydrogenase to couple chemiosmotic energy to hydrogen or formate production (Schmidt et al., 2013; Sieber et al., 2014).

Here, we apply proteomic and transcriptomic approaches to examine the role of SWOL_RS03525 and Hyd2*i*in syntrophic butyrate degradation by *S. wolfei*. We show that the SWOL_RS03525 and Hyd2 proteins were abundant in membranes of butyrate-grown *S. wolfei. hyd2* was up-regulated when *S. wolfei* was grown on butyrate with either *M. hungatei* or *Dehalococcoides mccartyi* strain 195 and SWOL_RS03525 was up-regulated when *S. wolfei* was grown on butyrate with *M. hungatei*. The abundance of SWOL_RS03525 and Hyd2 proteins and the up-regulation of their genes in butyrate-grown *S. wolfei* cells argue for their importance in reverse electron transfer during syntrophic butyrate degradation.

## Materials and Methods

### Strains and Cell Cultivation

*Syntrophomonas wolfei* subsp. *wolfei* strain Göttingen (DSM 2245B) was grown in axenic culture in an anoxic basal medium with 20 mM crotonate or in co-culture with *M. hungatei* strain JF1 (DSM 864 = ATCC 27890) with 20 mM crotonate or 10 mM butyrate in one liter of medium and incubated without shaking at 37°C (Sieber et al., 2014, 2015). Cultures were inoculated with 200 ml of the respective culture after a minimum of three transfers under the same growth conditions prior to cell harvest. Substrate utilization was monitored via high-pressure liquid chromatography (Sieber et al., 2014).

Co-cultures of *S. wolfei* and *Dehalococcoides mccartyi* strain 195 (ATCC BAA-2266 = KCTC 15142) were initially established in 160-ml serum bottles containing 100 ml of defined medium (He et al., 2007) with trichloroethylene (TCE) supplied at a liquid concentration of 0.6 mM (corresponding to 78 μmol trichloroethylene per bottle), 10 mM crotonic acid, and 100 μg/L vitamin B_12_ with a 90% N_2_:10% CO_2_ headspace at 34°C without shaking. A 5% (vol/vol) inoculation of each bacterium was used to establish the co-culture. *S. wolfei–D. mccartyi* co-cultures were subsequently transferred onto defined medium as described previously (Mao et al., 2015). Limitation of electron donor was achieved by reducing the butyrate concentration to 0.25 mM while keeping the amount of trichloroethylene constant (78 μmol per bottle). The purity of all cultures was routinely checked by phase-contrast microscopy.

### RNA Extraction, RNA Purification, cDNA Synthesis and qRT-PCR

For *S. wolfei–M. hungatei* transcript studies, after 50% substrate loss, which corresponded to the mid-exponential phase of growth, triplicate cultures were rapidly cooled in a dry ice-ethanol bath and centrifuged anoxically at 8000 ×*g* for 15 min. The cell pellet was resuspended in 1.5 ml of RNAlater (Applied Biosystems/Ambion, Austin, TX, USA) and then stored at -80°C until all the cultures were collected. Total RNA was obtained by using a RNeasy Mini Kit (Qiagen Inc., Valencia, CA, USA) as previously described (Sieber et al., 2014).

For *S. wolfei–D. mccartyi* transcript studies, triplicate axenic culture- and co-culture-grown cells were collected at late exponential phase (day 10 of the subculture) when 75% of the TCE was dechlorinated and about 20 μmol TCE remained in the co-culture. Cells were collected by vacuum filtration filter (60 mL culture per filter). Each filter was placed in a 2 mL orange-cap micro-centrifuge tube, frozen with liquid nitrogen and stored at -80°C until further processing.

The quality of all RNA samples was checked by electrophoresis and the concentrations of RNA samples were quantified by using a nano-photometer (IMPLEN, Westlake Village, CA, USA). Locus tag specific primers were designed using primer-BLAST and checked against *S. wolfei, M. hungatei* and *D. mccartyi* genome sequences (Supplementary Table S1). RNA was verified to be free of DNA contamination by PCR without reverse transcriptase. Desalted primers were made by Life Technologies (Carlsbad, CA, USA).

qRT-PCR was performed on biological triplicates with technical duplicates using the Bio-Rad MyIQ real-time PCR system and the iScriptT One-Step RT-PCR Kit with SYBR Green (Bio-Rad) for *S. wolfei–M. hungatei* samples as previously described (Sieber et al., 2014). For the *S. wolfei–D. mccartyi* transcript studies, cDNA was synthesized in 40 μL reaction mixes containing 50 ng RNA template, 0.5 μM concentration of random hexamer, and 50 U of reverse transcriptase by using a TaqMan reverse transcription reagent kit (Applied Biosystems, Foster City, CA, USA). cDNA samples from the reverse transcriptions were diluted fivefold with nuclease-free water and were quantified in three replicate qPCR reactions using SYBR fast mix. Amplification efficiency was determined by testing the primers against decreasing concentrations of DNA and the values can be found in Supplementary Table S1. The expression level of the target gene was normalized to the expression level of a reference gene, the DNA gyrase gene (Pfaﬄ, 2001).

### Blue-Native Polyacrylamide Gel Electrophoresis (BN-PAGE)

All culture manipulations were performed in the anaerobic chamber, and all centrifuge steps were done with sealed, anoxic, centrifuge tubes. *S. wolfei* axenic cultures and *S. wolfei–M. hungatei* co-cultures were harvested at 50 to 70% of the substrate by centrifugation (Sieber et al., 2014). Cells of *S. wolfei* were separated from those of *M. hungatei* by anaerobic Percoll density gradient centrifugation as previously described (Beaty et al., 1987; Sieber et al., 2014). Fractions containing less than 1% *M. hungatei* cells were pooled, diluted 500-fold in 50 mM potassium phosphate buffer (pH 7.2), and centrifuged at 7,000 ×*g* for 20 min at 4°C to remove residual Percoll.

Percoll-separated *S. wolfei* cells were resuspended in 4 ml of anoxic lysis buffer containing 20 mM 2,2-Bis(hydroxymethyl)-2,2′,2″-nitrilotriethanol (Bis-tris), 500 mM 𝜀-aminocaproic acid, 20 mM NaCl, 10 mM ethylenediaminetetraacetic acid (EDTA) and 10% (vol/vol) glycerol (pH 7.2; Swamy et al., 2006) and broken by passage through a French pressure cell at an internal pressure of 138,000 kPA (Sieber et al., 2014). Membrane fractions were obtained by ultracentrifugation as described previously (Sieber et al., 2014). The final pellet was resuspended in approximately 250 μl of anoxic lysis buffer containing 0.5% n-dodecyl-β-maltoside (DDM) to solubilize membrane proteins. Protein quantification was done using the Pierce BCA assay. Aliquots (25 μl) of solubilized membranes were stored frozen at -20°C in sealed microcentrifuge tubes.

BN-PAGE analysis was conducted using 4 or 16% acrylamide solutions (37.5:1 acrylamide:bis-acrylamide), each of which contained 50 mM Bis-tris and 67 mM 𝜀-aminocaproic acid (pH 7.2; Schagger and von Jagow, 1991; Swamy et al., 2006). The 16% acrylamide solution also contained 20% (vol/vol) glycerol. Polymerization was initiated with the separate addition of 10% ammonium persulfate and tetramethylethylenediamine in a 10:1 ratio (Schagger and von Jagow, 1991; Swamy et al., 2006). A gradient gel was immediately prepared using a mechanical gradient mixer and allowed to polymerize for 2 h. The cathode buffer contained 1.5 mM Bis-tris, 5.0 mM tricine, and 0.002% Coomassie blue G250 (w/v; pH of 7.0) and the anode buffer contained 5.0 mM Bis-tris (pH of 7.0; Swamy et al., 2006; Vizcaino et al., 2016). Solubilized membranes (2–35 μg protein) were mixed with an equal volume of sample buffer, containing 1 ml of cathode buffer, 7 ml of nanopure water, and 2 ml of electrophoresis grade glycerol. Gels were run at constant voltage (130 V) until the dye front migrated to within a few millimeters of the gel bottom. Gels were fixed and destained in a 50% methanol (v/v) and 7% acetic acid (v/v) solution, washed twice with nanopure water, and then stained with either Imperial stain (ThermoFisher), SilverStain (Pierce) or SyproRuby (ThermoFisher) according to manufacturer’s instructions.

### Tryptic Digest of BN-PAGE Membrane Complexes

Protein bands were manually excised and washed in a solution of 50 mM sodium bicarbonate and 50% acetonitrile and then in 100% acetonitrile. This procedure was repeated three times. Each gel slice was incubated at 60°C for 1 h in 10 mM dithiothreitol, then in 50 mM iodoacetamide at 45°C for 45 min in the dark, followed by washing three times with alternating solutions of 100 mM sodium bicarbonate and 100% acetonitrile. Each slice was dried and then incubated with 20 ng/μl porcine trypsin (Promega, Madison, WI, USA) for 45 min at 4°C, followed by incubation for 4–6 h in the same solution at 37°C. The digested protein was transferred into a fresh tube and each gel slice was extracted three times with a 10-min incubation in 50% acetonitrile:1% trifluoroacetic acid. The solutions with the extracted peptides and the initial peptide digestion solution were combined and then dried using a rotary evaporator at 30°C.

Peptide sequencing was accomplished with a nano-liquid chromatography (LC) tandem mass spectrometer (nano LC-MS/MS; QSTAR Pulsar XL, Applied Biosystems, Foster City, CA, USA) equipped with nanoelectrospray interface (Protana, Odense, Denmark) and LC Packings nano-LC system (Sunnyvale, CA, USA) with a homemade precolumn (150 mm × 5 mm) and an analytical column (75 mm × 150 mm) packed with Jupiter Proteo C12 resin (particle size 4 mm, Phenomenex, Torrance, CA, USA). Dried peptides were resuspended in 1% formic acid and six microliters were injected. The peptides were eluted at a flow rate of 200 nl/min using a gradient of 0.1% formic acid (solvent A) and 95% acetonitrile containing 0.1% formic acid (solvent B) as follows: 3 to 35% B for 72 min, 35 to 80% B for 18 min, then 80% B for 9 min. The precolumn was washed with 0.1% formic acid for 4 min before the sample was injected. The column was washed with 3% B for 15 min prior to the next run. Electrospray ionization was performed using a 30 mm (internal diameter) nanobore stainless steel online emitter (Proxeon, Odense, Denmark) at 1900 V. Peptide sequences were searched against the NCBI genomes for *S. wolfei, Syntrophus aciditrophicus* and *M. hungatei* using MASCOT software version 2.1 (Matrix Science, London, UK). The search against *S. aciditrophicus* genome was a contamination check as *S. aciditrophicus* is also cultured in our laboratory. Peptides were required to have a rank = 1, a score >18 and at least 2 unique peptides identified per protein. The maximum peptide false discovery rate was 5%. The proteomics data have been deposited in the PRIDE repository^1^ (Vizcaino et al., 2016) with the dataset identifier PXD003633.

### In-Gel Activity Staining

All manipulations were performed in the Coy anaerobic chamber. Precast 4–16% NativePage gels from Life Technologies were used with anaerobically prepared anode and cathode buffers. The lysis buffer was prepared and boiled under 80% N_2_: 20% CO_2_ for 5 min to remove oxygen. Stock solutions of heat labile components (e.g., 𝜀-aminocaproate and EDTA) were prepared in an anaerobic chamber using anoxic water. Six nanograms per lane of membrane protein suspension were electrophoretically separated as described. After electrophoresis, the gels were cut vertically and gel slices were placed in stoppered 100-ml Schott bottles and 20 ml of reaction buffer (50 μM benzyl viologen, 1 mM triphenyl tetrazolium chloride in 50 mM potassium phosphate pH 7.2) was added to each bottle. The bottles were stoppered and taken out of the anaerobic chamber. The headspace of the bottles with formate or no electron donnor was changed to 80% N_2_:20% CO_2_. Bottles with formate as the electron donor received 1 mM formate (final concentration) in 50 mM potassium phosphate (pH 7.2) while those with no electron donor and 80% N_2_:20% CO_2_ gas phase served as controls. Bottles with hydrogen as the electron donor had the gas phase of the anaerobic chamber (about 1% hydrogen and the balance nitrogen). Activity was monitored visually by the formation of a reddish-purple precipitate. A band testing positive for hydrogenase was manually excised and sent for tandem mass spectrometry by the proteomics core facility at the Oklahoma Medical Research Foundation. Full methods are available at: http://research.ouhsc.edu/CoreFacilities/MassSpectrometryProteomics.aspx.

## Results

### Routes for Reversed Electron Transfer in *S. wolfei*

To identify membrane proteins potentially involved in reverse electron transfer in *S. wolfei*, we performed blue native polyacrylamide gel electrophoresis (BN-PAGE) of solubilized membrane proteins from cells grown in axenic culture and co-culture with *M. hungatei* on crotonate, and in co-culture with *M. hungatei* on butyrate. Crotonate-grown cells do not require reverse electron transfer in contrast to butyrate-grown cells, which do. Several BN-PAGE protein bands were more pronounced in membranes prepared from butyrate-grown *S. wolfei* cells relative to membranes of *S. wolfei* cells grown on crotonate (**Supplementary Figures S1A,B**). One of these bands (band A34) contained the polypeptides of two subunits of the membrane-bound hydrogenase, Hyd2 (HydA2 and HydC2 encoded by SWOL_RS09950 and SWOL_RS09960, respectively; **Table 1**, **Supplementary Figure S1A**). Band B12 had a migration pattern similar to band A34 but showed some smearing. Band B12 contained polypeptides of Hyd2 (HydA2 and HydB2, the latter encoded by SWOL_RS09955) along with polypeptides of proteins annotated as a membrane-bound, iron-sulfur oxidoreductase (SWOL_RS03525 gene product), the Etf subunit, EtfB2 (SWOL_RS03515 gene product), SWOL_RS00720 gene product, and the alpha subunit of ATP synthase (SWOL_RS12360 gene product; **Table 1**; **Supplementary Figure S1B**). Likely, the smearing caused some overlap of protein migration patterns. Band A32 contained the iron-sulfur oxidoreductase polypeptide (the SWOL_RS03525 gene product; **Supplementary Figure S1A**) and bands with a similar migration pattern were detected in membranes from crotonate-grown *S. wolfei* cells (Supplementary Table S2). Hyd2 is the only membrane-bound hydrogenase predicted from genomic analysis (Sieber et al., 2010). However, the *S. wolfei* genome encodes genes for two membrane-bound formate dehydrogenases, *fdh2* (locus tags: SWOL_RS04025, SWOL_RS04030, SWOL_RS04035 and SWOL_RS04040) and *fdh4* (locus tags: SWOL_RS05200, SWOL_RS05205, SWOL_RS05210, SWOL_RS05215, SWOL_RS05220, and SWOL_RS05225). Interestingly, the four subunits of Fdh2 were detected in axenic culture *S. wolfei* cell membrane preparations (Bands A6 and A10, **Supplementary Figure S1**; Supplementary Table S2).

**Table 1:** Proteins detected in membrane protein bands that were more prominent when *S. wolfei* was grown syntrophically on butyrate.

Band	Locus tag	Protein	Number of unique peptides	Score
A34	SWOL_RS09950	HydA2	5	196
	SWOL_RS09960	HydC2	2+	33
B12	SWOL_RS09950	HydA2	41	1208
	SWOL_RS09955	HydB2	10	379
	SWOL_RS03515	EtfB2	2	151
	SWOL_RS03525	FeS oxidoreductase	2	106
	SWOL_RS12360	ATP synthase (alpha)	10	197
	SWOL_RS00720	S layer protein	6	311

### Enzyme Activity Staining

The solubilized membrane fractions of *S. wolfei* cells grown on butyrate were electrophoretically separated using BN-PAGE and the gels were subsequently incubated in the presence of tetrazolium red with either hydrogen or formate as the electron donor. Both conditions resulted in the reduction of tetrazolium red in solution after overnight incubation. A red precipitate was observed was on gels with hydrogen as the electron donor when 5 μg per ml of protein was used. The location of the precipitation coincided with the expected location band A34 (**Supplementary Figure S2**). Formate dehydrogenase activity was only observed when 20 μg of protein was used with an overnight incubation (data not shown). Peptide data showed that the band with hydrogenase activity contained all three subunits of the Hyd2 hydrogenase, Hyd2A, Hyd2B and Hyd2C (**Table 2**).

**Table 2:** Proteins detected in a membrane complex testing positive for hydrogenase activity in membrane fractions of *S. wolfei* grown on butyrate^a^.

Locus tag	Protein	Unique peptides	Score
SWOL_RS09950	HydA2	11	1043
SWOL_RS09955	HydB2	6	586
SWOL_RS09960	HydC2	1	61
SWOL_RS12600	Unknown function	1	171
SWOL_RS12350	ATP synthase (beta)	1	79

*^a^The band indicated in **Supplementary Figure S2** was excised and sent for tandem mass spectrometry to identify the proteins contained in the band.*

### Differential Expression of Reverse Electron Transfer Genes

**FIGURE 1:**
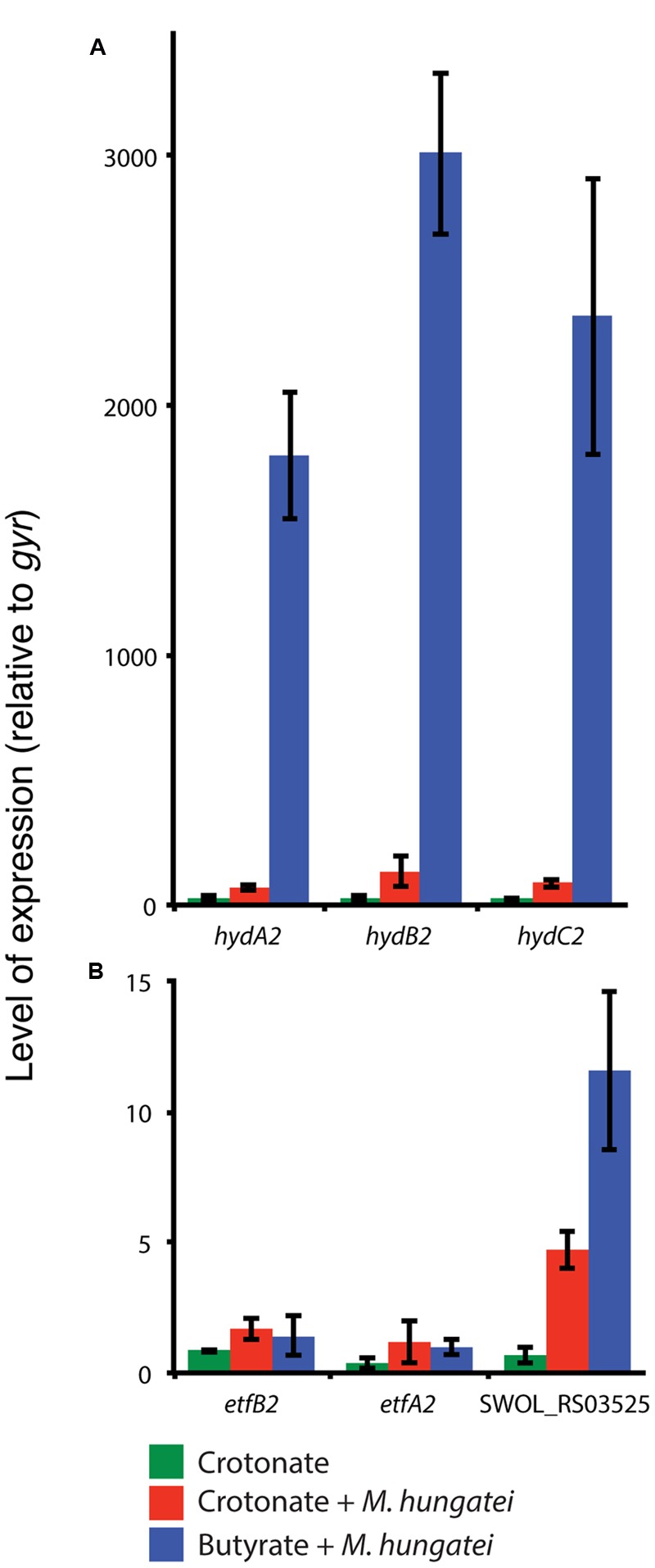
**Expression of *hydABC2* (SWOL_RS09950, SWOL_RS09955 and SWOL_RS09960) (A)**, *etfAB2* (SWOL_RS03515 and SWOL_RS00520) and SWOL_RS03525 (FeS oxidoreductase) **(B)** genes in *S. wolfei* grown in axenic culture on crotonate (green), in co-culture with *M. hungatei* on crotonate (red) or in co-culture with butyrate (blue).

We next performed qRT-PCR-based gene expression experiments to determine the relative transcript levels for the genes encoding the above mentioned polypeptides for *S. wolfei* Hyd2 hydrogenase, EtfAB2 and iron-sulfur oxidoreductase during axenic culture versus co-culture conditions with *M. hungatei*. Transcripts for each of the three *hyd2* genes, *hydA2, hydB2* and *hydC2* (SWOL_RS09950 SWOL_RS09955, and SWOL_RS09960, respectively) were significantly more abundant (ca. ∼ 20-fold) in *S. wolfei* cells grown syntrophically on butyrate compared to cells grown either in axenic culture or in co-culture on crotonate (**Figure 1**). Likewise, transcripts for the iron-sulfur oxidoreductase (SWOL_RS03525) were also elevated by 6- to 12-fold during co-culture growth on either butyrate or crotonate compared to axenic culture growth on crotonate (**Figure 1**). In contrast, expression of the *etfAB2* genes (SWOL_RS03515 and SWOL_RS03520) was not significantly changed under the three growth conditions (**Figure 1**).

**FIGURE 2:**
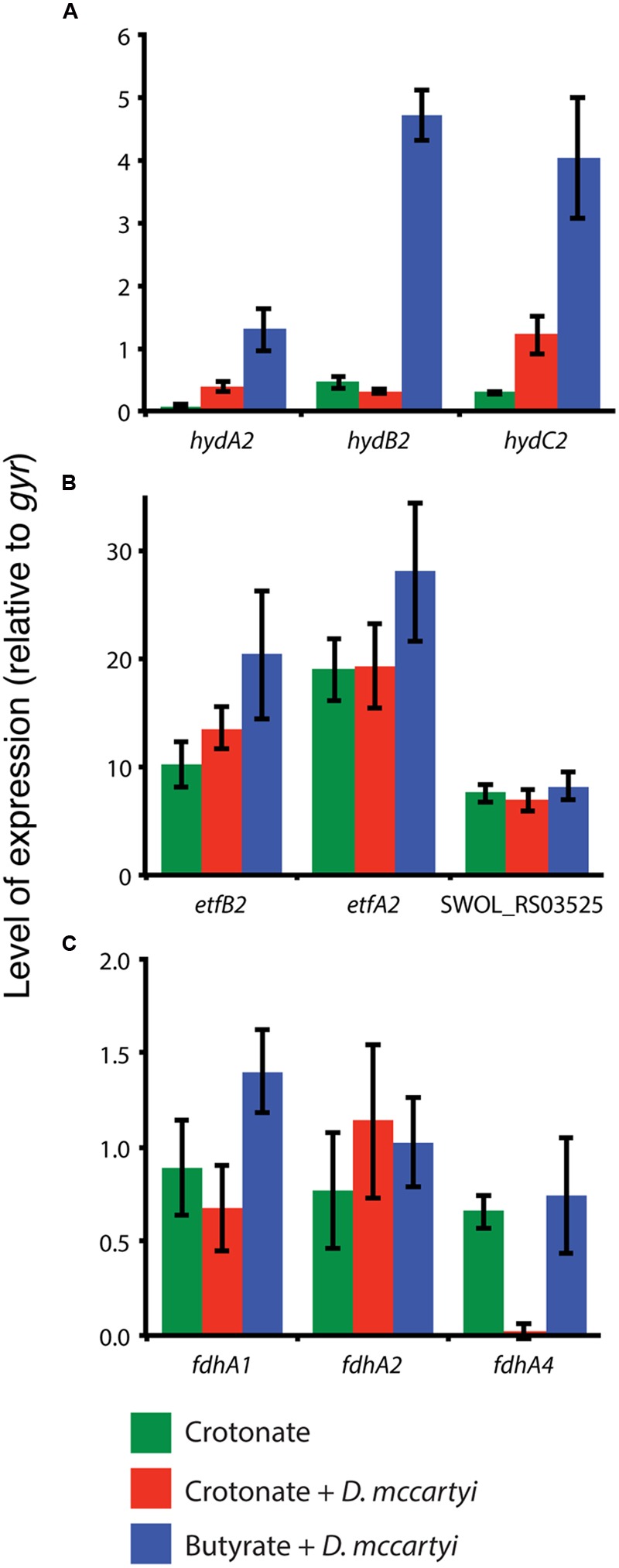
**Expression of *hydABC2* (SWOL_RS09950, SWOL_RS09955 and SWOL_RS09960) (A)**, *etfAB2* (SWOL_RS03515 and SWOL_RS00520) and SWOL_RS03525 (FeS oxidoreductase) **(B)** and formate dehydrogenase **(C)** genes in *S. wolfei* grown in axenic culture on crotonate (green), in co-culture with *D. mccartyi* on crotonate (red) or in co-culture with butyrate (blue).

The up-regulation of the *hyd2* genes implicates their importance for syntrophic butyrate degradation in *S. wolfei.* Since *M. hungatei* can use either hydrogen or formate for methanogenesis (Ferry et al., 1974), syntrophic butyrate metabolism may occur by either interspecies hydrogen or formate transfer from *S. wolfei* to *M. hungatei*. We therefore generated co-cultures that are dependent exclusively on interspecies hydrogen transfer (Materials and Methods). This was accomplished by culturing *S. wolfei s*yntrophically on butyrate with *D. mccartyi* strain 195 that can only use hydrogen for tetrachloroethene reduction (Maymo-Gatell et al., 1997; Mao et al., 2015). The expression of *hydA2* and *hydB2* in *S. wolfei* was 15- to 20-fold higher in cells grown syntrophically on butyrate with *D. mccartyi* relative to *S. wolfei* grown in co-culture on crotonate with *D. mccartyi* (**Figure 2**). The expression of *hydC2* was approximately the same in crotonate-grown and butyrate-grown co-culture cells and this level of expression was at least threefold higher than *hydC2* expression in crotonate-grown, axenic culture cells. The expression of the *S. wolfei etfA2* and *etfB2* genes was also slightly elevated in butyrate-grown cells with *D. mccartyi* relative to *S. wolfei* grown on crotonate in axenic culture or co-culture with *D. mccartyi* (**Figure 2**). Expression of SWOL_RS03525 was relatively unchanged under all three growth conditions. Lastly, the expression of the *S. wolfei fdhA1, fdhA2, fdhA4* genes did not change significantly (∼0.6- to 1.2-fold change) and was similar to that of the control gene, *gyrA*.

### Other Proteins Detected in *S. wolfei* Membranes

Native gel electrophoresis of the solubilized membrane proteins of *S. wolfei* revealed additional membrane-associated proteins (Supplementary Table S2). These included peptides derived from six of the subunits of the *S. wolfei* ATP synthase (α,β,γ,δ,b,c), three of which were detected under all growth conditions. Peptides of subunits of TRAP four-carbon dicarboxylate transport system (SWOL_RS00720 gene product), a branch-chain amino acid transporter (SWOL_RS13215 gene product), and six proteins with hypothetical annotations (gene products of SWOL_RS00720, SWOL_RS13360, SWOL_RS00780, SWOL_RS01665, SWOL_RS02325, and SWOL_RS10810) were detected under all growth conditions. One protein with a hypothetical annotation (SWOL_RS00720 gene product) was detected, which has been annotated as a copper amine oxidase protein, but has been recently suggested to be an S-layer protein (Schmidt et al., 2013). Other amino acid and inorganic ion transport proteins were detected in membranes of crotonate-grown, axenic culture *S. wolfei* cells (gene products of SWOL_RS01730, SWOL_RS09775, SWOL_RS02065, SWOL_RS10930, SWOL_RS012600 and SWOL_RS12825). It is unlikely that the above proteins serve a unique function during crotonate metabolism and likely rather serve in general cell metabolism.

A formate-nitrate transporter (SWOL_RS00525 gene product) and an inorganic pyrophosphatase (SWOL_RS05395 gene product) were detected in butyrate-grown cells. Schmidt et al. (2013) also detected the SWOL_RS00525 gene product only in *S. wolfei* cells grown with butyrate. Interestingly, another protein annotated as a succinate-acetate transporter (SWOL_RS09870 gene product) was detected only under co-culture conditions (Supplementary Table S2). The protein exhibits 25.4% identity and 43% similarity to the *Escherichia coli* YaaH transporter (b0010), which was recently demonstrated to function as a secondary transport system for proton-driven acetate uptake (Sá-Pessoa et al., 2013). This *S. wolfei* paralog could potentially operate physiologically in the reverse direction to expel acetate during syntrophic cell growth conditions, conditions where acetate production is high. This symport system would thereby aid in proton motive force generation across the cytoplasmic membrane to assist in the reverse electron transfer functions.

## Discussion

### Routes for Reversed Electron Transfer in *S. wolfei*

Several studies have hypothesized that the main conduit of electron transfer between acyl-CoA dehydrogenases and membrane redox carriers is through EtfAB2, and a membrane-bound iron-sulfur oxidoreductase (SWOL_RS03525 gene product; Müller et al., 2009; Schmidt et al., 2013; Sieber et al., 2015). We detected peptides of SWOL_RS03525 gene product under all growth conditions (**Table 1** and Supplementary Table S2), consistent with the findings of Schmidt et al. (2013). However, bands with the SWOL_RS03525 gene product were more pronounced in membranes of butyrate-grown *S. wolfei* cells (A12 and B32; Supplementary Table S2; **Supplementary Figure S2**), implicating its importance in syntrophic butyrate degradation. qRT-PCR studies supported the proteomic data showing that SWOL_RS03525 expression was elevated when *S. wolfei* was grown under conditions that required reverse electron transfer (i.e., butyrate growth with *M. hungatei*, **Figure 1**). However, the expression of SWOL_RS03525 was relatively unchanged when *S. wolfei* was grown with *D. mccartyi* with crotonate or butyrate compared to growth of *S. wolfei* alone on crotonate (**Figure 2**).

BN gels also showed that Hyd2 was differentially abundant in membranes of butyrate-grown *S. wolfei* cells (Supplementary Table S2; **Supplementary Figure S2**). Activity staining showed that this band had hydrogenase activity and was comprised of subunits of Hyd2 (**Table 2**). Gene expression studies again supported proteomic analysis that *hydABC2* was differentially expressed when *S. wolfei* was grown with either *M. hungatei* or *D. mccartyi* (**Figures 1** and **2**). Clearly, Hyd2 is important for syntrophic butyrate metabolism. Syntrophic growth of *S. wolfei* on butyrate in co-culture with *D. mccartyi* confirms that syntrophic butyrate degradation can occur exclusively by interspecies hydrogen transfer as *D. mccartyi* is unable to use formate for tetrachloroethene reduction (Maymo-Gatell et al., 1997; Mao et al., 2015) and that Hyd2 is an important enzyme in this process. This reliance on hydrogen may be growth condition dependent, as formate dehydrogenase activity is present and Fdh2 has been also implicated in interspecies electron transfer when the co-culture is grown with limited iron, no CoM, and supplemented with yeast extract at 30°C (Schmidt et al., 2013).

### Model for Reversed Electron Transfer in *S. wolfei*

**FIGURE 3:**
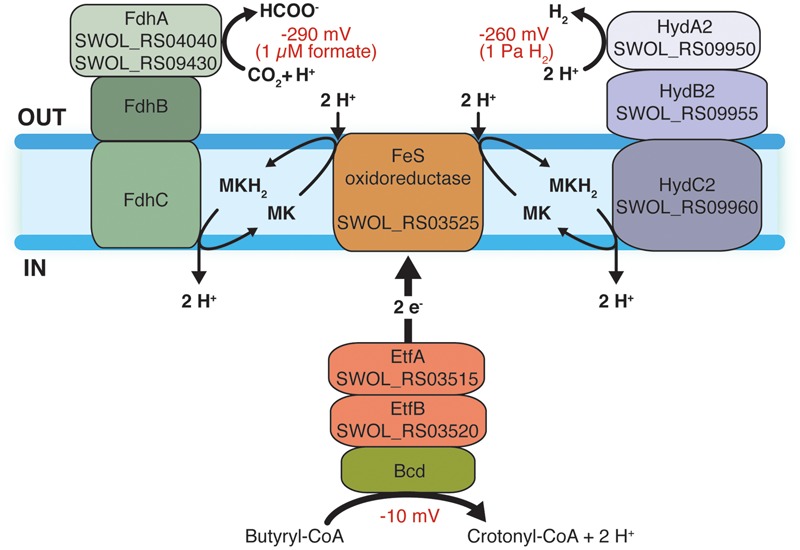
**Model for reverse electron transfer during syntrophic butyrate oxidation by *S. wolfei*.** Electrons from butyryl-CoA dehydrogenase are transferred to the iron-sulfur (FeS) oxidoreductase (SWOL_RS03525 gene product) by (EtfAB2, SWOL_RS03515 and SWOL_RS03520). FeS oxidoreductase and an [FeFe]-hydrogenase form separate complexes in the membrane. Electrons from FeS oxidoreductase reduce menaquinone with protons consumed at outside surface of the membrane. The [FeFe]-hydrogenase oxidizes menaquinol with the release of protons on the inside of the cell. Menaquinol oxidation could also occur by a membrane-bound formate dehydrogenase (Fdh). The consumption of protons during hydrogen or formate production and the inward flow of protons would drive the unfavorable redox change involved in hydrogen or formate production from electrons generated during the oxidation of butyryl-CoA. Redox values for butyryl-CoA oxidation at standard conditions and hydrogen and formate metabolism at 1 Pa and 1 μM, respectively, are given. Abbreviations: Bcd, butyryl-CoA dehydrogenase; ETF, electron transfer flavoprotein); Hyd, hydrogenase; Fdh, formate dehydrogenase; MK, menaquinone; and MKH_2_, menaquinol. Numbers in parentheses are locus tag designations of the respective genes.

Our proteomic and gene expression data support a model for reverse electron transfer during syntrophic butyrate oxidation by *S. wolfei* involving a quinone loop (**Figure 3**) where the membrane-bound, iron-sulfur oxidoreductase (SWOL_RS03525 gene product) acts as an EtfAB:menaquinone oxidoreductase to receive electrons from acyl-CoA dehydrogenases via Etf2 and subsequently reduce menaquinone to menaquinol (Sieber et al., 2010; Schmidt et al., 2013). Menaquinol is reoxidized by either a membrane-bound hydrogenase or a membrane-bound formate dehydrogenase (Schmidt et al., 2013) depending on the syntrophic mode of growth (Sieber et al., 2014). The translocation of protons by the quinone loop along with the consumption of protons on the outside of the membrane during hydrogen or formate production would supply the necessary chemiosmotic energy for reverse electron transfer (Schmidt et al., 2013). The driving force for reverse electron transfer would be the creation of a chemiosmotic potential by the ATP synthase hydrolyzing ATP formed by substrate-level phosphorylation reactions (Wofford et al., 1986). Additionally, YaaH-type transporter (SWOL_RS09870 gene product) could contribute to the creation of a chemiosmotic potential by coupling acetate excretion with proton translocation.

### Implications for Analysis of Metagenomics Data

Genomic and proteomic analyses have implicated a number of gene systems in syntrophic electron flow and hydrogen and formate production in *S. wolfei* (Schmidt et al., 2013; Sieber et al., 2014, 2015). The presence of these genes in metagenomics data for environmental samples has been used to implicate syntrophic hydrocarbon metabolism in these environments (Nobu et al., 2015; Oberding and Gieg, 2016; Wawrik et al., 2016). However, experimental evidence to support that role of various redox proteins, hydrogenases and formate dehydrogenases has been sparse. Previous studies provided support for *fdh2* (Schmidt et al., 2013) and *hyd2* (Sieber et al., 2014) in syntrophic butyrate metabolism. Here, we show that Hyd2 has hydrogenase activity and that SWOL_RS03525 gene product is more abundant and *hyd2* and SWOL_RS03525 are differentially expressed when *S. wolfei* is grown syntrophically on butyrate with either *M. hungatei* or *D*. *mccartyi* as the syntrophic partner. Clearly, Hyd2 plays an important role in syntrophic metabolism by *S. wolfei–D*. *mccartyi*, as butyrate degradation by this co-culture can only proceed via interspecies hydrogen transfer. *hydABC2* is also likely to be important for syntrophic microorganisms as these genes are present in the genomes of other known fatty acid-degrading syntrophic bacteria and *hydC2* is present in the draft genome sequence of *Desulfosporosinus* sp. Tol-M, which syntrophically metabolizes tolulene (Laban et al., 2015) (**Supplementary Figure S3**).

## Author Contributions

BC, JS, and XM designed and conducted experiments and wrote the manuscript; RO and HN conducted proteomic analyses and helped write the manuscript; and RG, LA-C, and MM helped designed experiments, analyze data, and write the manuscript.

## Conflict of Interest Statement

The authors declare that the research was conducted in the absence of any commercial or financial relationships that could be construed as a potential conflict of interest.

## Footnotes

1 http://www.ebi.ac.uk/pride/archive/

## Supplementary Material

FIGURE S1 **Blue native gels of solubilized membrane proteins of *S. wolfei* grown in axenic culture on crotonate and in co-culture with *M. hungatei* on crotonate or butyrate.** Two different sets of cultures were analyzed in **(A,B)**. Protein bands were numbered, excised, digested, and analyzed by mass spectroscopy. Proteins identified in each band are listed in Supplementary Table S2.

FIGURE S2 **Hydrogenase and formate dehydrogenase activity staining of membrane proteins from Percoll-separated *S. wolfei* cells grown on butyrate with *M. hungatei*.** The gel was sliced longitudinally and the slices were placed in anaerobic culture bottles with hydrogen, formate, or no electron donor added as indicated. Molecular weight markers are shown at the left. A red precipitate formed with hydrogen as donor, but not in the other incubations. The band with hydrogenase activity from a different gel was excised and analyzed (**Table 2**).

FIGURE S3 **Hyd2 in syntrophic, fatty acid-degrading and tolulene-degrading syntrophic bacteria.** The numbers are percentages of identity at the amino acid level to the respective *S. wolfei* gene product. NCBI accession numbers are given below each gene except for *Thermosyntropha lipolytica*, where Integrated Microbial Genomics locus tags are used as the amino acid coding sequences for *T. lipolytica* are not present in NCBI.
