# Supplementary material for: Membrane Complexes of Syntrophomonas wolfei Involved in Syntrophic Butyrate Degradation and Hydrogen Formation
Source: Front Microbiol. 2016 Nov 9;7:1795. doi: 10.3389/fmicb.2016.01795 (PMC5101538; doi:10.3389/fmicb.2016.01795)
Supplement: Supplementary file 1 [file Table_1.DOCX]

Supplemental Table 1: Primers for quantitative RT-PCR analysis of *hydABC2, etfAB2,* Swol_0698*, fdhA-1, fdhA-2 and fdhA-4* in *S. wolfei*.

| Gene designation | Locus Tag | Predicted localization | Predicted function | Forward Primer | Reverse Primer | Binding Efficiency |
| --- | --- | --- | --- | --- | --- | --- |
| *gyrB*^1^ | SWOL_RS03520 | cytoplasm | DNA gyrase | TGAAGGACAGACCAAAACCA | AATATAGCCTGGTAGGTGCG | 1.84 |
| *hydIIA*^1^ | SWOL_RS09950 | periplasm | MQ ↔ H_2_(Fe-H_2_ase) | TATGCGGAGGACAACTACCC | CTGAGGATTTCATAGGCGGT | 1.87 |
| *hydIIB* | SWOL_RS09955 | membrane | MQ ↔ H_2_(Fe-H_2_ase) | GAAAGTGAAGGCATCACCAG | GCTTCATGCACATAATGGGG | 1.71 |
| *hydIIC* | SWOL_RS09960 | membrane | Cytochrome B | CCAACGCCTCTCTAGTTCAT | AGAAATACTGCAGGCACAGA | 1.82 |
| *fdhA1*^1^ | SWOL_RS03970 | membrane | Formate ↔ MQ | CATAGAAGCCAACCGGGAAA | CCCTTCTCTCGGTGTTGGTA | 2.00 |
| *fdhA2*^1^ | SWOL_RS04040 | cytoplasm | Formate ↔ NADH | CAGCATCAGCAGCAAAAGAG | CTTCCCACTTGTCACTACCA | 1.73 |
| *fdhA4*^1^ | SWOL_RS05220 | cytoplasm | Formate ↔ NADH | GAATACCCGTTCCTGCTTTC | GCTTAACCGCACAGACCTTG | 1.71 |
| *etfA* | SWOL_RS03515 | cytoplasm | ETF-α | AATAGTGGTTGTTGCTGCTG | ACTTCGGTTACCTGAGTTCC | 1.99 |
| *etfB* | SWOL_RS03520 | cytoplasm | ETF-β | TGTTGCCAACTTCACATACG | TATGGTGCGGACAAGGTTTA | 2.05 |
| *FeS oxred* | SWOL_RS03525 | membrane | FeS oxidoreductase | ACCCTGGTAAGCCAGAACCT | TGGTCGTAGACCCCGTTGTG | 1.91 |

^1^ Sieber, J. R., H. Le and M. J. McInerney (2014). The importance of hydrogen and formate transfer for syntrophic fatty, aromatic and alicyclic metabolism. Environ. Microbiol. **16**: 177-188. doi: 110.1111/1462-2920.12269.
