# Supplementary material for: Membrane Complexes of Syntrophomonas wolfei Involved in Syntrophic Butyrate Degradation and Hydrogen Formation
Source: Front Microbiol. 2016 Nov 9;7:1795. doi: 10.3389/fmicb.2016.01795 (PMC5101538; doi:10.3389/fmicb.2016.01795)
Supplement: Supplementary file 2 [file Table_2.DOCX]

Supplemental Table 2. Proteins detected in blue-native gels of solubilized membranes of *S. wolfei* grown in pure culture and coculture with *M. hungatei*.

| Locus tag | NCBI protein identification number^1^ | IMG Locus Tag^1^ | Functional annotation | Detected under the following growth conditions | | |
| --- | --- | --- | --- | --- | --- | --- |
|  |  |  |  | Crotonate pure culture | Crotonate co-culture | Butyrate co-culture |
| **Amino acid transport and metabolism** | | | | | | |
| SWOL_RS01170 | WP_011639682 | Swol_0222 | Hypothetical protein | +^2^ |  |  |
| SWOL_RS02090 | WP_011639856 | Swol_0410 | (R) 2-hydroxyacyl-CoA dehydratase, beta subunit | + |  |  |
| SWOL_RS03255 | WP_011640076 | Swol_0644 | Tryptophan synthase, beta subunit | + |  |  |
| SWOL_RS04085 | WP_008516151 | Swol_0809 | 3-deoxy-D-arabinoheptulosonate-7-phosphate synthase | + |  |  |
| SWOL_RS06975 | WP_011640755 | Swol_1348 | 3-deoxy-7-phosphoheptulonate synthase | + |  |  |
| SWOL_RS10930 | WP_049750190 | Swol_2113 | ABC-type branched-chain amino acid transporter, periplasmic subunit | + |  |  |
| SWOL_RS11085 | WP_011641526 | Swol_2144 | Ketol-acid reductisomerase | + |  |  |
| SWOL_RS13215 | WP_011641923 | Swol_2556 | ABC-type branched-chain amino acid transporter, periplasmic subunit | + | + | + |
| **Carbohydrate transport and metabolism** | | | | | | |
| SWOL_RS01685 | WP_011639780 | Swol_0331 | TRAP-type C4 transporter, periplasmic subunit | ++ | + | + |
| SWOL_RS02065 | WP_015735797 | Swol_0405 | ABC-type sugar transporter, periplasmic subunit | ++ |  |  |
| **Cell wall/membrane/envelope biogenesis** | | | | | | |
| SWOL_RS08950 | WP_041427498 | Swol_1734 | Hypothetical protein/4-hydroxybutyrate:acetyl-CoA CoA transferase (EC 2.3.1.-) | + |  |  |
| SWOL_RS13815 | WP_011641843 | Swol_2472 | Peptidase M23B (Murein DD-endopeptidase MepM and murein hydrolase activator NlpD, contain LysM domain) |  | + |  |
| **Coenzyme transport and metabolism** | | | | | | |
| SWOL_RS04115 | WP_011640241 | Swol_0815 | Phenylacetate-CoA ligase | ++ |  |  |
| **Defense mechanisms** | | | | | | |
| SWOL_RS00935 | WP_011639638 | Swol_0176 | Acridine resistance plasma membrane protein | + |  |  |
| **Energy production and conversion** | | | | | |  |
| SWOL_RS03515 | WP_041427355 | Swol_0696 | Electron transfer flavoprotein, beta subunit |  |  | + |
| SWOL_RS03525 | WP_011640127 | Swol_0698 | Iron-sulfur oxidoreductase | ++ | + | ++ |
| SWOL_RS04025 | WP_011640223 | Swol_0797 | Formate dehydrogenase, gamma subunit | + |  |  |
| SWOL_RS04030 | WP_011640224 | Swol_0798 | Formate dehydrogenase, iron-sulfur subunit | + |  |  |
| SWOL_RS04035 | WP_041427376 | Swol_0799 | Formate dehydrogenase, major subunit | + |  |  |
| SWOL_RS04040 | WP_041427377 | Swol_0800 | Formate dehydrogenase, major subunit | + |  |  |
| SWOL_RS05395 | WP_041427823 | Swol_1064 | Inorganic diphosphatase |  |  | + |
| SWOL_RS06135 | WP_011640607 | Swol_1195 | Indolepyruvate oxidoreductase subunit beta 2 | + |  |  |
| SWOL_RS09870 | WP_011641301 | Swol_1911 | Hypothetical protein (Succinate-acetate transporter protein) |  | + | + |
| SWOL_RS09950 | WP_011641315 | Swol_1925 | Ferredoxin hydrogenase, Hyd2, alpha subunit |  |  | ++ |
| SWOL_RS09955 | WP_011641316 | Swol_1926 | Ferredoxin hydrogenase, Hyd2, beta subunit |  |  | + |
| SWOL_RS09960 | WP_011641317 | Swol_1927 | Ferredoxin hydrogenase, Hyd2, gamma subunit |  | + | + |
| SWOL_RS10985 | WP_011641507 | Swol_2124 | FixC: electron transfer flavoprotein-quinone oxidoreductase | + |  |  |
| SWOL_RS12350 | WP_011641755 | Swol_2382 | ATP synthase, beta subunit | ++ | ++ | + |
| SWOL_RS12355 | WP_011641756 | Swol_2383 | ATP synthase, gamma subunit | + | + |  |
| SWOL_RS12360 | WP_011641757 | Swol_2384 | ATP synthase, alpha subunit | ++ | ++ | ++ |
| SWOL_RS12365 | WP_011641758 | Swol_2385 | ATP synthase F1 subunit delta | + | + |  |
| SWOL_RS12370 | WP_011641759 | Swol_2386 | ATP synthase, subunit b | ++ | ++ | + |
| SWOL_RS12375 | WP_041427598 | Swol_2387 | ATP synthase, subunit c | ++ | ++ |  |
| **Function Unknown** | | | | | | |
| SWOL_RS00720 | WP_041427240 | Swol_0133 | Hypothetical protein (S layer homology domain) | ++ | + | ++ |
| SWOL_RS13360 | WP_011639607 | Swol_0141 | Hypothetical protein | + | ++ | ++ |
| SWOL_RS00780 | WP_011639609 | Swol_0143 | Hypothetical protein | ++ | ++ | ++ |
| SWOL_RS01665 | WP_011639775 | Swol_0325 | Hypothetical protein (ABC-type uncharacterized transport system, periplasmic component) | + | + | + |
| SWOL_RS02110 | WP_011639859 | Swol_0413 | Hypothetical lipoprotein | + | + |  |
| SWOL_RS02325 | WP_011639900 | Swol_0457 | Hypothetical protein | + | + | + |
| SWOL_RS02850 | WP_011640000 | Swol_0562 | Hypothetical protein (Alkaline shock protein Asp23 ) |  | + |  |
| SWOL_RS05945 | WP_011640574 | Swol_1161 | Protein of unknown function UPF0182 | + | + |  |
| SWOL_RS06080 | WP_011640597 | Swol_1185 | LemA protein | + | ++ | + |
|  | WP_011641155 | Swol_1762 | Hypothetical protein | + |  |  |
| SWOL_RS10810 | WP_011641472 | Swol_2089 | Hypothetical protein | ++ | + | + |
| **Inorganic ion transport and metabolism** | | | | | | |
| SWOL_RS00525 | WP_011639559 | Swol_0091 | Formate/nitrite transporter |  |  | + |
| SWOL_RS01730 | WP_011639790 | Swol_0341 | ABC-type nitrate/sulfonate/bicarbonate transport system, periplasmic component | + |  | + |
| SWOL_RS09775 | WP_011641283 | Swol_1891 | Cation ABC transporter, periplasmic binding protein | + |  |  |
| SWOL_RS12600 | WP_011641805 | Swol_2432 | ABC-type metal ion transport system, periplasmic component/surface antigen | + | + |  |
| SWOL_RS12825 | WP_011641847 | Swol_2476 | Iron (III)-binding transporter, periplasmic protein | + |  |  |
| **Intracellular trafficking, secretion, and vesicular transport** | | | | | | |
| SWOL_RS03450 | WP_011640114 | Swol_0682 | Sec-independent protein translocase protein TatA | + |  |  |
| SWOL_RS07365 | WP_011640830 | Swol_1424 | Protein translocase subunit secF | ++ | + | + |
| SWOL_RS07370 | WP_011640831 | Swol_1425 | Protein-export membrane protein SecD | ++ | + | + |
| **Lipid transport and metabolism** | | | | | | |
| SWOL_RS02095 | WP_011639857 | Swol_0411 | Activator (R)-2-Hydroxyacyl-CoA dehydratase | + |  |  |
| SWOL_RS02235 | WP_011639882 | Swol_0436 | 4-Hydroxyburyrate coenzyme A transferase | + |  | + |
| SWOL_RS03415 | WP_011640107 | Swol_0675 | Acetyl-CoA acetyltransferase | + |  |  |
| SWOL_RS09995 | WP_011641324 | Swol_1934 | Acetyl-CoA acetyltransferase |  |  | + |
| SWOL_RS10495 | WP_011641416 | Swol_2030 | 3-hydroxyacyl-CoA dehydrogenase | ++ |  |  |
| **Unassigned** | | | | | | |
| SWOL_RS09665 | WP_049750127 | Swol_1871 | Glycosyl hydrolase-like protein | + | + | + |
| SWOL_RS13250 | WP_011641930 | Swol_2563 | YyaC (putative sporulation protein YyaC) |  | + |  |
| **Posttranslational modification, protein turnover, chaperones** | | | | | | |
| SWOL_RS02485 | WP_011639931 | Swol_0490 | Chaperone protein DnaK | + | + |  |
| SWOL_RS08165 | WP_011640979 | Swol_1577 | Chaperone DnaJ |  | + |  |
| SWOL_RS08570 | WP_049750120 | Swol_1659 | Hypothetical protein | + |  |  |
| SWOL_RS09590 | WP_011641248 | Swol_1855 | Chaperonin GroEL (HSP60 family) | ++ | + | + |
| SWOL_RS11200 | WP_011641549 | Swol_2167 | Membrane Protease protein family | + |  |  |
| **Replication, recombination and repair** | | | | | | |
| SWOL_RS05845 | WP_011640556 | Swol_1142 | ATP-dependent nuclease, subunit B | + |  |  |
| SWOL_RS08930 | WP_011641125 | Swol_1730 | Exonuclease |  | + | + |
| **Secondary metabolites, transport, and catabolism** | | | | | | |
| SWOL_RS01440 | WP_011639735 | Swol_0278 | Esterase/lipase |  | + | + |
| **Signal Transduction** | | | | | | |
| SWOL_RS04670 | WP_011640348 | Swol_0927 | Methyl-accepting chemotaxis sensory transducer | + |  | + |
| **Translation, ribosomal structure and biogenesis** | | | | | | |
| SWOL_RS12025 | WP_011641696 | Swol_2321 | LSU ribosomal protein L5P |  | + | + |
| SWOL_RS12095 | WP_011641710 | Swol_2335 | 50S ribosomal protein L29 | + |  |  |
| SWOL_RS12135 | WP_011641718 | Swol_2343 | 50S ribosomal protein L7/L12 | + |  |  |

^1^ Abbreviations: NCBI, National Center for Biotechnology Information; IMG, Integrated Microbial Genomes

^2^ +, detected in one of the replicate; ++, detected in both replicates
