# Supplementary figures and images for: Membrane Complexes of Syntrophomonas wolfei Involved in Syntrophic Butyrate Degradation and Hydrogen Formation
Source: Front Microbiol. 2016 Nov 9;7:1795. doi: 10.3389/fmicb.2016.01795 (PMC5101538; doi:10.3389/fmicb.2016.01795)

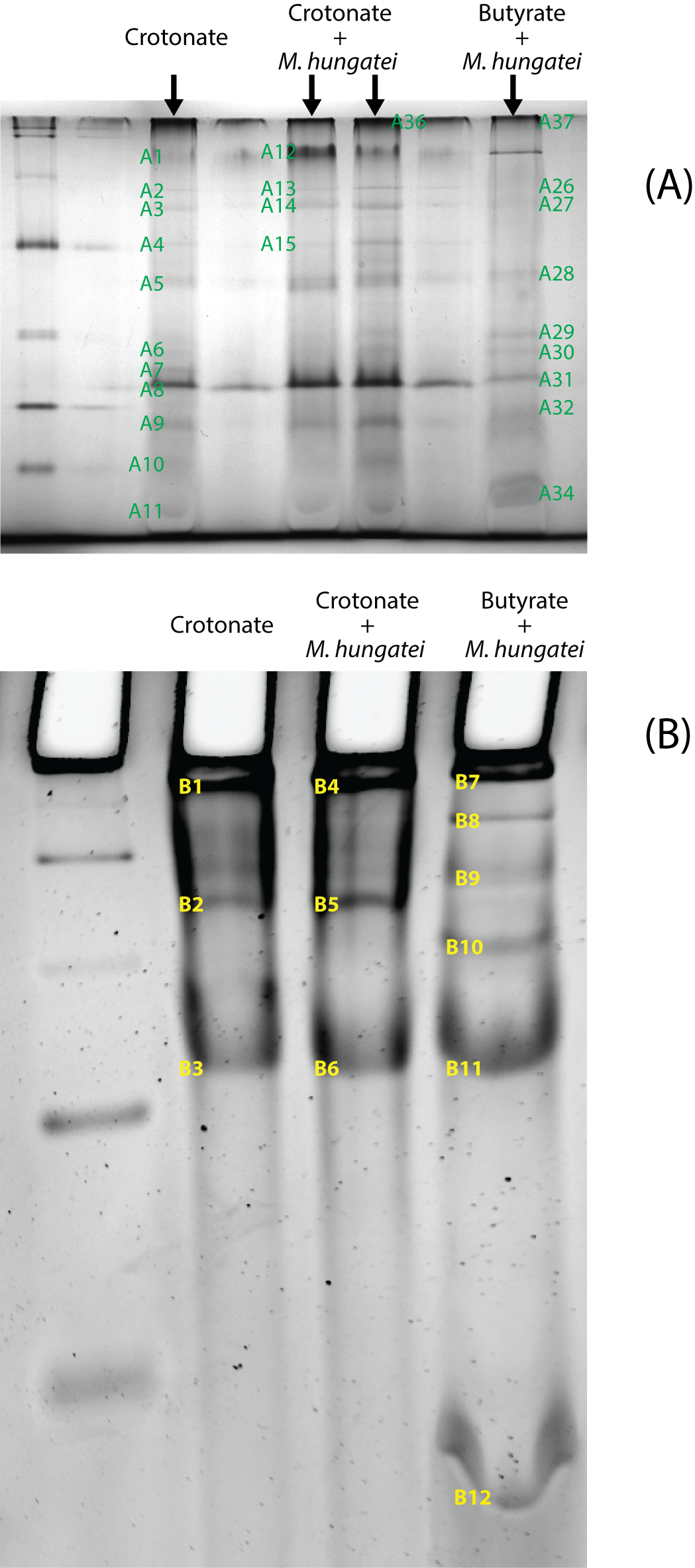

Supplement: FIGURE S1 — Blue native gels of solubilized membrane proteins of S. wolfei grown in axenic culture on crotonate and in co-culture with M. hungatei on crotonate or butyrate. Two different sets of cultures were analyzed in (A,B). Protein bands were numbered, excised, digested, and analyzed by mass spectroscopy. Proteins identified in each band are listed in Supplementary Table S2. [file Image_1.TIF]

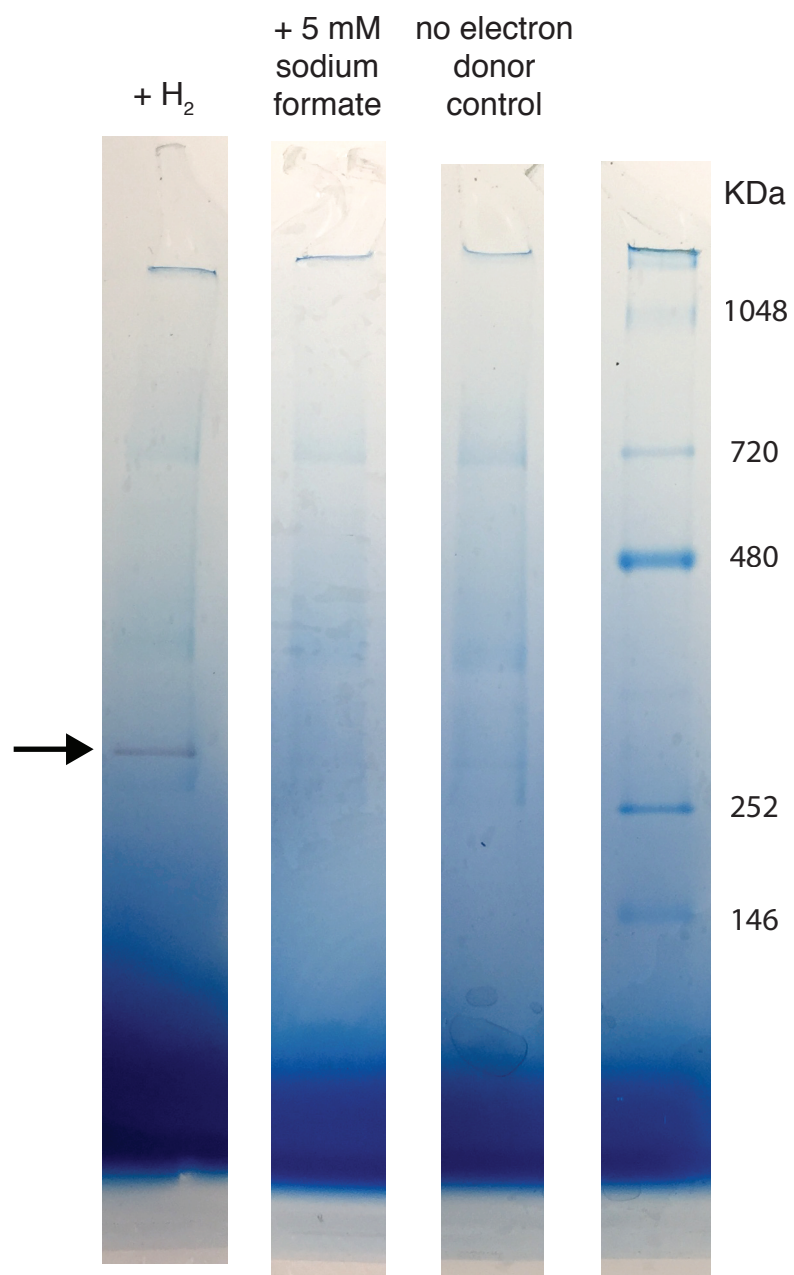

Supplement: FIGURE S2 — Hydrogenase and formate dehydrogenase activity staining of membrane proteins from Percoll-separated S. wolfei cells grown on butyrate with M. hungatei. The gel was sliced longitudinally and the slices were placed in anaerobic culture bottles with hydrogen, formate, or no electron donor added as indicated. Molecular weight markers are shown at the left. A red precipitate formed with hydrogen as donor, but not in the other incubations. The band with hydrogenase activity from a different gel was excised and analyzed (Table 2). [file Image_2.PDF]

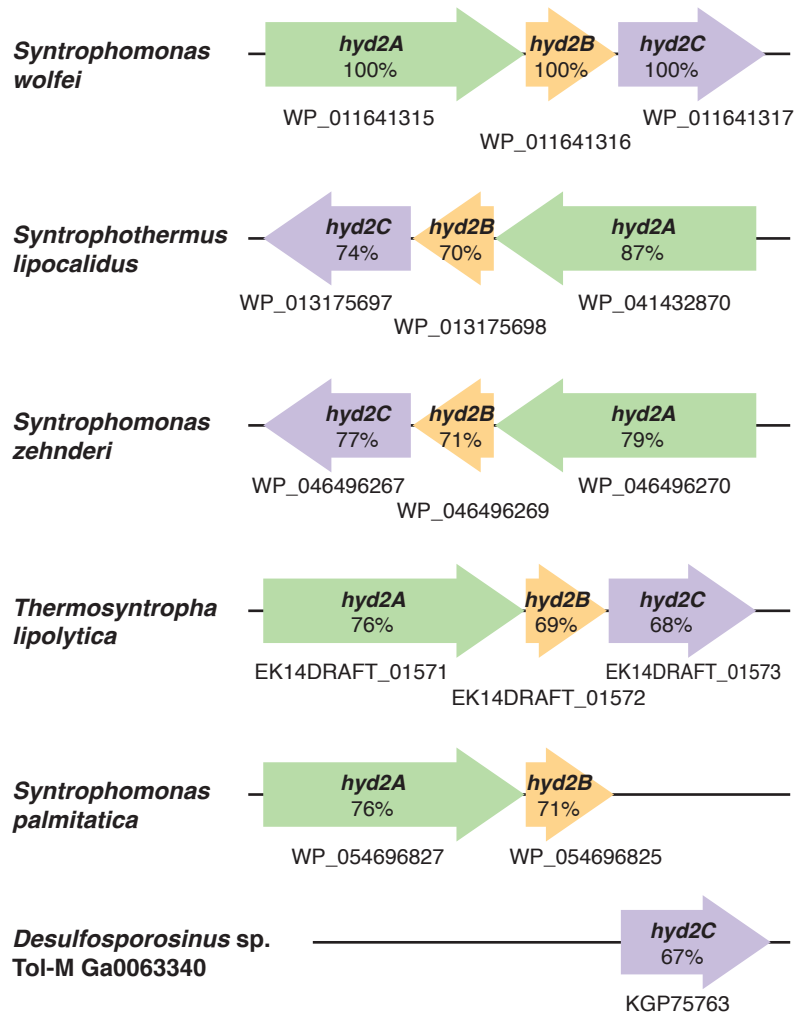

Supplement: FIGURE S3 — Hyd2 in syntrophic, fatty acid-degrading and tolulene-degrading syntrophic bacteria. The numbers are percentages of identity at the amino acid level to the respective S. wolfei gene product. NCBI accession numbers are given below each gene except for Thermosyntropha lipolytica, where Integrated Microbial Genomics locus tags are used as the amino acid coding sequences for T. lipolytica are not present in NCBI. [file Image_3.PDF]
